# The Roads to Haploid Embryogenesis

**DOI:** 10.3390/plants12020243

**Published:** 2023-01-05

**Authors:** Kun Shen, Mengxue Qu, Peng Zhao

**Affiliations:** 1 State Key Laboratory of Hybrid Rice, College of Life Sciences, Wuhan University, Wuhan 430072, China; 2 Hubei Hongshan Laboratory, Wuhan 430070, China

**Keywords:** parthenogenesis, haploid induction, single fertilization, genome elimination, crop breeding

## Abstract

Although zygotic embryogenesis is usually studied in the field of seed biology, great attention has been paid to the methods used to generate haploid embryos due to their applications in crop breeding. These mainly include two methods for haploid embryogenesis: in vitro microspore embryogenesis and in vivo haploid embryogenesis. Although microspore culture systems and maize haploid induction systems were discovered in the 1960s, little is known about the molecular mechanisms underlying haploid formation. In recent years, major breakthroughs have been made in in vivo haploid induction systems, and several key factors, such as the matrilineal (MTL), baby boom (BBM), domain of unknown function 679 membrane protein (DMP), and egg cell-specific (ECS) that trigger in vivo haploid embryo production in both the crops and Arabidopsis models have been identified. The discovery of these haploid inducers indicates that haploid embryogenesis is highly related to gamete development, fertilization, and genome stability in ealry embryos. Here, based on recent efforts to identify key players in haploid embryogenesis and to understand its molecular mechanisms, we summarize the different paths to haploid embryogenesis, and we discuss the mechanisms of haploid generation and its potential applications in crop breeding. Although these haploid-inducing factors could assist egg cells in bypassing fertilization to initiate embryogenesis or trigger genome elimination in zygotes after fertilization to form haploid embryos, the fertilization of central cells to form endosperms is a prerequisite step for haploid formation. Deciphering the molecular and cellular mechanisms for haploid embryogenesis, increasing the haploid induction efficiency, and establishing haploid induction systems in other crops are critical for promoting the application of haploid technology in crop breeding, and these should be addressed in further studies.

Although zygotic embryogenesis is the primary way to generate embryos in plant reproduction, there are still several alternative methods for forming haploid or diploid embryos. In addition to normal zygotic embryogenesis, in recent decades, a great deal of attention has been paid to the formation of haploid embryos due to their important applications in crop breeding. Haploid induction is an effective way to shorten breeding time and has been used in crop breeding for many years. Traditional breeding requires 7–8 generations to obtain ideal homozygous plants during the cross, while haploid technology can shorten the breeding time to 2–3 generations, which will greatly save on breeding time and reduce the cost of breeding [1]. Haploid embryogenesis is the core of haploid breeding technology.

According to the existing methods of generating haploid embryos, a haploid induction system can be divided into two categories: in vitro and in vivo methods. Microspore embryogenesis is the primary in vitro method used to induce haploid embryos. It was first developed in 1964 and has been employed in over 75 species. The in vitro methods for haploid embryogenesis are mainly achieved through the suitable in vitro cell culture system, which promotes haploid microspores to be reprogrammed and enter into embryogenic pathways to generate haploid embryos. However, these in vitro culture systems have not been established for many plant species, and the mechanisms of microspore embryogenesis remain largely unknown. The existing in vivo haploid induction systems can be further divided into CENH3-mediated haploid embryogenesis, parental factor-induced haploid embryogenesis, and transcriptional factor-triggered haploid embryogenesis. The haploid inducer line in maize was first discovered in 1959, but the mechanisms underlying haploid formation have long remained a mystery. The recent discoveries of several key molecular players in maize and Arabidopsis haploid induction have greatly aided us in understanding the mechanisms of haploid embryogenesis, demonstrating that defects in gamete development and fertilization are the fundamental mechanisms for haploid generation.

In this review, we summarize the recent advances in the methods for generating haploid embryogenesis, and we primarily focus on microspore embryogenesis, CENH3-mediated haploid embryogenesis, and parental factor-induced and transcription factor-triggered haploid embryogenesis. We also discuss the mechanisms underlying these methods and the potential applications of haploid embryogenesis in crop breeding.

## 1. Microspore Embryogenesis

Microspore embryogenesis is a type of embryogenesis in which haploid microspores after stress treatment undergo cell reprogramming and shift into embryogenic pathways to generate haploid embryos [2,3,4,5,6,7,8]. The haploid embryos then can be automatically doubled or chemically treated to produce double haploids, thus reducing the time required to obtain homozygous plants [9,10]. Among these microspore embryogenesis systems, exine-dehisced *Brassica napus* microspores treated by physical stress can induce polarization and develop into typical embryos with the differentiation of an embryo proper and suspensor [11]. However, microspore embryogenesis induction systems have not been well established in many other plants, such as *Arabidopsis thaliana* and *Solanum lycopersicum*, indicating that the embryogenic potential of microspores or signals for triggering cell fate reprogramming into embryogenic pathways may vary in different species.

**Figure 1:**
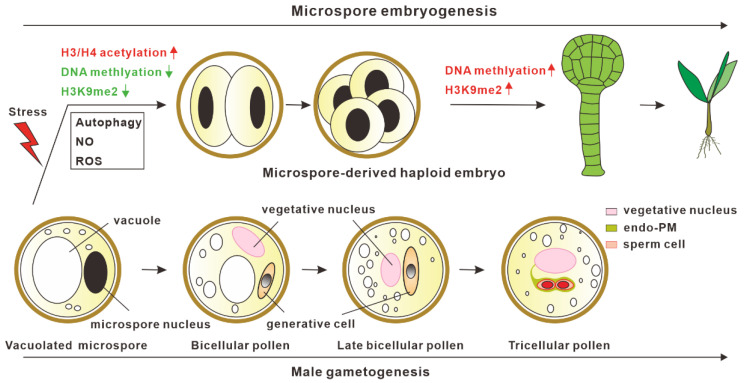
Model for in vitro microspore embryogenesis. After stress treatment, vacuolated microspores switch from normal male gametogenesis into the embryogenic pathway. Epigenetic modifications, autophagy, reactive oxygen species (ROS), and nitric oxide (NO) are involved in promoting the initiation of microspore embryogenesis.

In microspore embryogenesis, the reprogramming of microspores into the embryo developmental pathway after stress treatment is the critical step for haploid embryo generation. Epigenetic mechanisms, including H3/ H4 deacetylation, DNA methylation, and H3K9me2, are reported to be involved in microspore embryogenesis [12]. The inhibition of histone deacetylases (HDAC) activities by the chemical inhibitor Trichostatin A (TSA) can efficiently promote microspore embryogenesis in both *B. napus* and *A. thaliana*. Suppression of HDAC activities by TSA leads to the hyperacetylation of histones H3 and H4, which results in the upregulation of genes related to cell wall remodeling, cell division, and embryogenesis [13]. In addition to *B. napus* and *A. thaliana,* TSA treatment has been efficient at improving the rate of microspore embryogenesis in several other plants, including pakchoi [14], wheat [15,16], and barley [17], indicating that the HDAC-dependent mechanism in microspore embryogenesis appears to be conserved in different plants. Besides suppressing HDAC activities, the inhibition of DNA methyltransferases catalytic activities by the inhibitors 5-azacytidine (AC) and 2′-deoxy-5-azacytidine (DAC) can remarkably increase the frequency of microspore embryogenesis [18], suggesting that decreased DNA methylation levels are also responsible for microspore embryogenesis, which is likely achieved through increased chromatin accessibility for transcription activation. Similar results in promoting microspore embryogenesis were also observed when the microspores were treated with BIX-01294, which can efficiently inhibit the activities of H3K9me2 methyltransferase [19]. In summary, a low DNA methylation level, low H3K9me2 level, and high acetylation level are critical for the initiation of microspore embryogenesis, whereas, after reprogramming, microspore embryogenesis is accompanied by increased DNA methylation levels and H3K9me2 levels [19,20] at later developmental stages, which may promote haploid embryo differentiation (Figure 1).

Besides epigenetic modifications, autophagy, reactive oxygen species (ROS), and nitric oxide (NO) have been shown to be involved in the initiation process of microspore embryogenesis (Figure 1) [12,21,22]. Recent studies have revealed that autophagy-mediated cytoplasm clearance is not only critical for promoting pollen germination [23] and pollen tube growth [24], but it is also important for microspore embryogenesis [25,26,27]. Two independent studies have demonstrated that the cell death of microspores after stress treatment is accompanied by the activation of autophagy, and blocking autophagy prevented the cell death of microspores and increased the frequency of microspore embryogenesis in *B. napus*, indicating that enhanced autophagic activities play a role in preventing microspore embryogenesis [27]. A similar role of autophagy in *Hordeum vulgare* microspore embryogenesis was also found. The suppression of autophagosome formation by 3-methyladenine or the inhibition of autophagic body degradation in the vacuoles by E-64 can promote microspore embryogenesis [26]. In addition, ROS and NO were also shown to play important roles in response to stress-induced microspore cell death and microspore reprogramming in barley microspore embryogenesis [28]. Stress-treated microspore embryonic suspension exhibited high ROS levels, high NO signals, and enhanced microspore cell deaths [28,29]. Treating microspores with MnCl_2_ (O_2_^−^ scavenger), ascorbate (H_2_O_2_ scavenger), and cPTIO (NO scavenger) led to reduced cell death and increased embryogenesis initiation efficiency. Hence, investigating these mechanisms will not only help us understand the mechanism for microspore embryogenesis initiation, but also provide an opportunity to improve haploid induction.

## 2. CENH3-Mediated Haploid Embryogenesis

CENH3 (CENP-A in humans, Cse4p in *Saccharomyces cerevisiae*, and HTR12 in *Arabidopsis*) [30,31] is the centromere-specific histone H3 variant which contains a variable N-terminal domain (NTD) and a conserved histone fold domain (HFD). The core functions of CENH3 primarily include two aspects: recruiting CENH3/H4 reloading factor to nucleosomes and providing a platform for kinetochore binding and assembling. The NTD of CENH3 is essential for CENH3 loading onto the centromeres of meiotic chromosomes, rather than its deposition and function in mitotic nuclei [32,33]. Recent studies have revealed that both the quality (stability of CENH3 on nucleosomes and recognizability of CENH3 by CENH3/H4 reloading factors or kinetochore complex proteins) and quantity (loading amount of CENH3 nucleosomes on centromeres) of CENH3 in centromeres are important for CENH3′s function [34,35,36,37,38,39], and defects in CENH3 will lead to chromosome elimination.

Based on the characteristics of CENH3, GFP-CENH3 and GFP-tailswap (GFP fused with the N-terminal tail of a CENH3 variant, with NTD replaced by conserved Histone3.3 NTD) were designed to rescue the developmental defects in a *cenh3-1* mutant. Expressing *GFP-tailswap* in a *cenh3-1* mutant can partially recuse the embryonic lethal phenotype, but it is accompanied by severe male sterility [33,40]. More importantly, expressing *GFP-tailswap* or *GFP-CENH3* in a *cenh3-1* mutant can produce aneuploid and haploid plants due to chromosome elimination. When *GFP-tailswap/cenh3-1* pistils were pollinated by wild-type (WT) pollen grains, over 30% of the progenies were haploids, though with paternal genomes. Haploid-induced rates (HIR) slightly decreased when *GFP-tailswap* expressing pollen grains were used for the cross with the WT plants. Expressing *CENH3* from *B. rapa*, *Lepidium oleraceum*, or *Z. mays* in the Arabidopsis *cehn3-1* mutant can also lead to haploid formation, which is similar to the expression *GFP-CENH3* or *GFP-tailswap* in *cenh3-1* [41]. In addition, point mutations in CENH3 can also induce haploids during the outcrossing with WT plants [39,42,43]. Under this scenario, the CENH3-mediated haploid induction system appears to be conserved in different plants.

**Figure 2:**
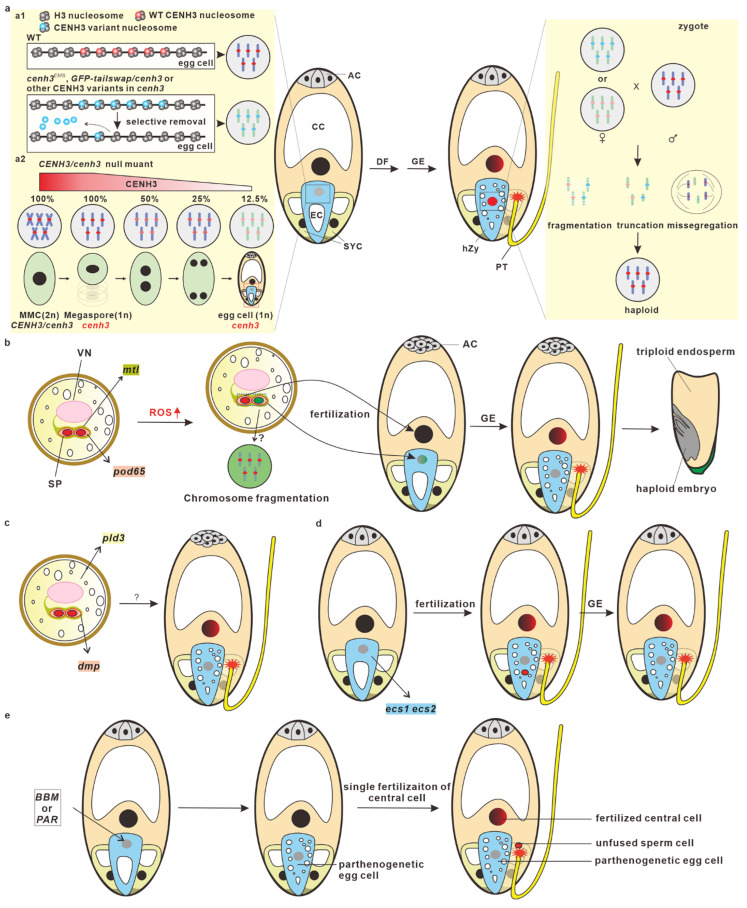
Model of in vivo haploid embryogenesis. (**a**) CENH3-mediated haploid embryogenesis is accomplished through post-fertilization genome elimination. This model shows CENH3-mediated maternal genome elimination after fertilization. In a *cenh3* *^EMS^* mutant, the *GFP-tailswap*/*cenh3* mutant or *cenh3* mutants which express the CENH3 variant from other species, selectively eviction of CENH3 variants in mature egg cells and the zygotes produce “weak” centromeres (**a1**). In a heterozygous model of a *cenh3* null mutant, the quantity of CENH3 on the centromeres is significantly diluted after megasporogenesis, which also generates “weak” centromeres in *cenh3* egg cells (**a2**). After being pollinated by wild-type pollen grains, the maternal genome (gray), which contains “weak” centromeres, rather than the normal paternal genome (red), is selectively eliminated in zygotes and early embryos. (**b**) Mutations in *MTL* and *POD* induces haploid embryogenesis through ROS-triggered spermatid chromosome fragmentation. MTL is localized at the endo-PM of vegetative cells while POD65 is localized in sperm cells. An ROS level increase leads to spermatid chromosome fragmentation. Sperm cells (green) with fragmented chromosomes can fertilize egg cells, but the paternal genomes will be eliminated after fertilization (gray). (**c**) Vegetative cell-expressed *PLD3* and sperm cell-expressed *DMP* may induce maternal haploid embryogenesis. (**d**) Mutation in egg cell-specifically expressed *ECS1/2* induces haploid embryogenesis, which may be due to the karyogamy defect and genome elimination after fertilization. (**e**) The ectopic expression of *BBM* and *PAR* in egg cells induces haploid embryogenesis through parthenogenesis. WT, wild-type; MMC, megaspore mother cell; AC, antipodal cell; CC, central cell; EC, egg cell; SYC, synergid cell; hZy, haploid zygote; DF, double fertilization; GE, genome elimination; PT, pollen tube.

The potential mechanisms underlying CENH3-mediated haploid induction have been summarized and discussed, and several hypotheses and theories have been proposed in recent reviews [36,38,44,45,46,47,48,49,50,51,52]. CENH3-induced haploids are the result of post-fertilization genome elimination [53]. Based recent published results, fertilization is normally completed to form zygotes, but uniparental genomes will be eliminated at early embryonic developmental stages, and eventually they will form the haploid mature embryos. Mutations or modifications in CENH3 may impair the recruitment of CENH3 to centromeres or reduce their stability, resulting in the selective dispossession of CENH3 variants from centromeres in egg cells and zygotes [54]. In a heterozygous *cenh3* null mutant, the CENH3 amount on the centromeres of the *cenh3* embryo sac are significantly diluted during post-meiotic cell division, prior to gamete formation [55]. The inconsistency of the centromere strength caused by CENH3 among the two parental genomes in zygotes thereby leads to the delay of CENH3 reloading and/or kinetochore assembly in uniparental genomes, resulting in chromosome missegregation, truncation, or fragmentation. Chromosomal irregularities can produce micronuclei or lead to the missegregation of chromosomes [56]. The chromosomal truncation and fragmentation may form micronuclei, which may re-emerge with the nucleus to produce aneuploids or eventually degrade to produce haploid embryos (Figure 2a).

## 3. Parental Factor-Mediated Haploid Embryogenesis

Although the paternal inducer line of maize (Stock6), which can be used to induce maternal haploids, was discovered in 1959 [57], the specific gene responsible for haploid generation was not identified until recently. In this section, we will summarize the advances in identifying both maternal and parental factors that could induce haploid embryos and discuss the mechanism underlying these parental factors in haploid embryogenesis.

### 3.1. Paternal Players in Haploid Embryogenesis

Four paternal factors (*MTL*/*PLA1*/*NLD*, *DMP*, *PLD3*, and *POD65*) have been identified from maize (Figure 2b,c). *RMZM2G47124*, the first-identified maternal haploid-inducing gene cloned from *qhir1*, was named *MATRILINEAL* (*MTL*) [58], *PHOSPHOLIPASE A1* (*PLA1*) [59], and *NOT LIKE DAD* (*NLD*) [60] by three different groups. MTL/PLA1/NLD (hereafter referred to as MTL) belongs to the phospholipase family and is an enzyme that hydrolyzes the phospholipids that function in membrane remodeling [61,62,63]. MTL is localized at the pollen endo-plasma membrane, a special plasma membrane that originates in the plasma membrane of vegetative cells and closely surrounds two sperm cells [64]. A 4 bp insertion leads to a frame-shift mutation in the *MTL*, which results in seed abortion and haploid induction. The second-identified maternal haploid inducer gene is *ZmDMP*, which encodes a DUF679 domain membrane protein and is specifically expressed in sperm cells. *ZmDMP* mutation induced an HIR of 0.1~0.3%, but it significantly increased HIR at a five-to-six-fold in combination of *MTL* mutation [65]. In addition to maize, recent studies have demonstrated that the role of *DMP* in haploid induction is conserved between monocots and eudicots, which has been achieved in *A. thaliana*, *B. napus* [66,67], *B. oleracea* [68], *Medicago truncatula* [69], *Nicotiana tabacum* [67,70], *S. lycopersicum* [71], and *S. tuberosum* [72]. In addition to *MTL* and *DMP*, vegetative cell-expressed *ZmPLD3* and sperm cell-expressed *ZmPOD65* were also shown to be able to induce haploids in maize [73,74]. ZmPLD3 belongs to phospholipase D (PLD) family, and it is localized in the ER, plastids, the Golgi apparatus, and the cytosol of vegetative cells [73], whereas *ZmPOD65* encodes a peroxidase (POD) protein and is highly expressed in pollen at the tricellular stage [74].

### 3.2. Maternal Factor in Haploid Embryogenesis

In addition to the paternal factors in haploid embryogenesis, recent reports have demonstrated that egg cell-expressed maternal factors can also be used to induce haploid embryos. *EGG CELL-SPECIFIC1/2* (*ECS1*/2) encodes egg cell-specifically expressed aspartic proteases, which are secreted to the synergid cell region upon fertilization to avoid polytubey by degrading the pollen tube attractant LURE1 [75]. Recently, two independent studies demonstrated that the mutation of *ECS1* and *ECS2* can also induce haploid embryogenesis [76,77]. Unfused sperm nuclei were observed in zygotes and early embryos, suggesting that karyogamy defects occurred in the sperm–egg fertilization, and the haploids from the *ecs1 ecs2* mutant progenies may have resulted from the post-fertilization genomic elimination [77]. In summary, *ECS*-mediated haploid induction is likely caused by pseudogamy and potential post-fertilization genomic elimination (Figure 2d).

### 3.3. Synergistic Effects on Haploid Embryogenesis

Since a low efficiency of haploid embryogenesis is observed in most inducer lines, efficiency has become a major barrier for its application in crop breeding. To improve the efficiency of haploid embryogenesis, the combination of *MTL* and *DMP* [65] or *MTL* and *PLD3* [73] was performed to test whether they could improve haploid production. The mutation of *PLD3* or *DMP* in *mtl* mutant background could significantly increase its haploid induction rate, but the HIR was still lower than the expected. A *dmp*–*mtl*–*pld3* triple mutant was also created to test whether it could increase the HIR. However, *dmp*–*mtl*–*pld3* triple-homozygous plants could not be obtained, which was likely due to the pollen developmental defect or the fertilization defect in triple mutants. Hence, it is still worth testing other combinations of haploid inducer genes to improve the efficiency of haploid induction in further studies.

### 3.4. Mechanism for Haploid Embryogenesis

Two mechanisms have been proposed to explain the haploid formation in maize. The first is a single fertilization-induced haploid and the second is post-fertilization genome elimination. The former may produce haploid embryos through the parthenogenesis of the egg cell while the central cell fertilizes normally to form the endosperm. In the latter, double fertilization occurs normally, but the zygote undergoes uniparental genome elimination that results in haploid embryo formation.

The mechanisms for haploid embryogenesis are primarily focused on *MTL*-induced haploid embryogenesis, and whether it is conserved among different inducers, such as *DMP*, remains largely unknown. Several recent studies have demonstrated that *MTL*-induced haploids may form through post-fertilization genome elimination, rather than single fertilization-mediated parthenogenesis. The markers, including B chromosomes and CENH3-YFP derived from the paternal genome, were detected in haploid progenies [78], suggesting that the egg cells were fertilized successfully, and that uniparental genome elimination occurred during haploid induction. In addition, when WT pistils were pollinated by *mtl* mutant pollen grains (which were carrying the *Cas9* and *gRNA* expression cassette) [79], genome-edited haploids without the *Cas9* expression cassette were detected in the progenies. Since the CRISPR/*Cas9* system only exists in the paternal genome, this result strongly suggests that the paternal genome is transmitted to the egg cell upon fertilization and is eventually eliminated after fertilization. A multi-omics analysis of *mtl* pollen grains revealed that ROS signals were involved in post-fertilization genome elimination [74]. Elevated ROS levels may cause DNA damage in pollen from the *mtl* mutant [80], which may lead to chromosome fragmentation in sperm cells and then induce genome elimination after fertilization. Spermatid chromosome fragmentation in the CAU5 haploid inducer line was detected through single nucleus sequencing [81]. In addition, the in vitro treatment of pollen grains with ROS-inducing reagents can also result in sperm DNA fragmentation and lead to the formation of haploids when pollinated to the WT plants. Studies on the sperm cell-expressed peroxidase gene *ZmPOD65* further confirmed the role of ROS in haploid embryogenesis [74]. Peroxidases wildly exist in the plant kingdom [82], and they convert hydrogen peroxide (H_2_O_2_) into H_2_O during the POD catalytic reaction [83]. Therefore, POD65 may be functional in the removal of H_2_O_2_ in sperm cells, and *POD65* mutation can cause H_2_O_2_ to burst in sperm cells, which leads to sperm DNA fragmentation and, eventually, haploid production.

In summary, vegetative cell-expressed *MTL* and sperm cell-expressed *POD65* are involved in the regulation of ROS levels, as well as the expression of ROS-related genes in pollen grains. Mutations in *MTL* and *POD65* do not appear to impair fertilization, but the zygotes generated from the cross between *mtl* or *pod65* mutants and WT plants will undergo genome elimination to form haploid embryos (Figure 2b). An *MTL* mutation may cause an imbalance in the hydrolyzed phospholipids (such as PC) between sperm cells and vegetative cells, resulting in the over-accumulation of PCs in sperm cells, which disrupts mitochondrial homeostasis and leads to increased ROS levels [84]. Elevated ROS levels in sperm cells will induce DNA damage and impair the expression of ROS-related genes, leading to the chromatin fragmentation. The fragmented chromatins of sperm cells are then eliminated after fertilization, leading to the formation of haploid embryos. This genome elimination mechanism is different from that in CENH3-mediated haploid embryogenesis. In the *MTL*- or *POD65*-induced haploid embryos, chromosome fragmentation occurs before fertilization and the fragmented paternal genome cannot function properly after fertilization and is eventually eliminated, whereas the CENH3-induced post-fertilization genome elimination is largely due to the incompatibility of the parental genome.

## 4. Transcription Factors Triggered Haploid Embryogenesis

Normal zygotic embryogenesis is triggered by the fusion of a sperm cell and an egg cell. However, recent reports have demonstrated that egg cells can bypass fertilization to trigger embryogenesis by expressing the transcriptional factors BABY BOOM (BBM) or PARTHENOGENESIS (PAR). BBM, an AINTEGUMENTA-like (AIL) transcription factor, belongs to the APETALA2/ETHYLENE RESPONSE FACTOR (AP2/ERF) family, which was previously shown to play a critical role in inducing or enhancing somatic embryogenesis [85,86,87,88,89,90,91,92]. In addition to triggering somatic embryogenesis, recent studies have demonstrated that the ectopic expression of *BBM* in egg cells can trigger egg cells to initiate embryogenesis in multiple sexual plants [93,94,95,96,97,98,99], indicating the conserved role of BBM in promoting embryogenesis. For example, in rice, the ectopic expression of *BBM1* in egg cells can produce embryo-like structures without fertilization [96]. An expression pattern analysis revealed that *OsBBM1* is expressed in sperm cells, and only the paternal allele of *OsBBM1* is expressed in 2.5 HAP (hours after pollination) zygotes. Both the maternal and paternal transcripts could be detected in 6.5 HAP zygotes, possibly due to the auto-activation ability of BBM. Recently, the ectopic expression of *BnBBM* in the egg cells of *A. thaliana*, *B. napus*, and *S. lycopersicon* also bypassed fertilization for embryogenesis [99], although how *BnBBM* triggered the egg cells to initiate the embryo program remains unknown. Besides *BBM*, *PAR* has also been reported to be able to induce parthenogenesis in sexual plants (Figure 2e). *PAR* encodes a zinc finger domain protein with an EAR motif, which may function as a transcription factor in dandelion (*Taraxacum officinale*). The ectopic expression of *ToPAR* in an egg cell can also promote that egg cell to produce embryo-like structures without fertilization in sexually reproductive lettuce [100]. There are two *PAR* homologs, named *DAZ3* and *TREE1,* in the *Arabidopsis* genome. It is worth investigating whether the homologs of *PAR* can also trigger egg cells to initiate embryogenesis in other plants, especially in crops.

## 5. Application of Haploid Embryogenesis

### 5.1. Haploid Breeding

The main application of haploid embryogenesis is to generate haploid plants which can accelerate the gain of homozygous plants and efficiently shorten breeding times. Most haploid induction systems have been tested in multiple crops (Table 1). The CENH3-mediated haploid induction system can be used to generate both maternal and paternal haploids, whereas the other haploid induction systems discussed above can only produce maternal haploids. The CENH3-mediated paternal haploid induction method can also be used to introduce a nuclear genome of interest into the targeted cytoplasm, such as the cytoplasmic male sterile (CMS) line. Therefore, the CENH3-mediated haploid induction system is also a useful tool for the establishment or improvement of the CMS line for hybrid seed production. However, CENH3-mediated haploid induction systems have not been successfully used in crop breeding [49], primarily due to the low HIR in crops and the complex steps required to construct CENH3 induction lines. In addition, the *MTL*-mediated haploid induction system has only been validated in monocots [58,59,60,79,80,101,102,103,104], while the *DMP*-mediated haploid induction system has been confirmed in both monocots and eudicots [65,66,67,68,69,70,71,72,105]. Hence, it is worth optimizing the haploid induction systems to promote their application in crop breeding in future studies.

### 5.2. Genome Editing

In addition to haploid breeding, a haploid induction system combined with CRISPR/Cas9 technology could be used for genome editing in crops that are resistant to genetic transformation. Gene-editing strategies, named haploid induction editing technology (HI-EDIT) and haploid inducer mediated genome editing (IMGE), have been developed based on in vivo haploid induction systems, respectively [79,108]. In the HI-EDIT system, CRISPR-Cas9 technology was combined with the *MTL*-mediated or CENH3-mediated haploid induction system to create a one-step genome editing method. Targeted genome loci could be efficiently edited in the haploid progenies and steadily inherited in the next generation, and at the same time, the *Cas9* construct could be completely removed through genome elimination. This method is based on the post-fertilization genome elimination of the haploid induction systems. In maize, when pollinated by *mtl* pollen grains that carry the *Cas9* construct, five of six maize germplasms received a more than 3% editing efficiency increase in the haploids via the HI-EDIT method. In *Arabidopsis*, a CENH3 haploid induction system, in combination with CRISPR-Cas9 technology, was developed for the HI-EDIT method. The results demonstrated that when *Arabidopsis Landsberg erecta* pollen grains pollinated to the Arabidopsis *cenh3-1* mutant (*Columbia* ecotype) expressing CENH3 from maize and the *Cas9* construct [109], 16.9% of the targeted genes in the haploid progenies were edited [79]. Thus, the HI-Edit method works effectively in both monocots and eudicots, which can provide a power tool for wide applications in genome-editing technology of the commercial variety. Similarly, Baobao Wang et al. [108] developed an approach, named IMGE, for genome editing. They introduced a CRISPR/Cas9 cassette into the CAU5 inducer line and tested its ability for genome editing in haploids, and they reported an approximately 4.1% increase in editing efficiency in the haploids. Hence, HI-EDIT and IMGE can be efficiently used for genome editing in crops that are resistant to genetic transformation, and their use avoids the interference of transgenes on crop traits through uniparental chromosome elimination.

### 5.3. Heterosis Fixation

Another application of haploid embryogenesis is for heterosis fixation, when used in combination with the *MiMe* (*Mitosis instead of Meiosis*) system [110,111,112]. In *MiMe* system, the combination of the mutations in the genes which are responsible for abolishing meiotic recombination, separating the sister chromatids and skipping the second division during meiosis, respectively, will shift meiosis into a mitosis-like division [113,114]. Since *MiMe* gametes are diploid, the introduction of a haploid induction system into the *MiMe* background can produce clonal propagation seeds with hybrid genotypes [96,102,115]. For example, *osd1–pair1–rec8–mtl* quadruple mutants (named *Fix*, for *Fixation of hybrids*) were used for heterosis fixation in rice [102]. Approximately 6.2% of the *Fix* diploid progenies with the same genotype as the mother were obtained, indicating that the *Fix* system can produce clonal seeds and fix heterozygosity in the F_1_ generation of hybrid rice. Recent results have demonstrated that the heterotic phenotypes and synthetic apomixis traits of these clonal seeds could be stably transmitted to the next generation [116]. Similarly, the *MiMe* system, in combination with the ectopic expression of *BBM1* in egg cells, was also successfully used for heterosis fixation in rice, and clonal seeds were obtained from 11% and 29% of the progeny of the two transgenic lines of the *MiMe* plus *BBM1-ee* (ectopic expression of *OsBBM1* in egg cells) constructs [96], respectively. In further studies, more attention should be paid to increasing the efficiency of clonal seeds.

## 6. Conclusions and Perspectives

There are two alternative roads for generating haploid embryos, as described above. Microspore embryogenesis can not only be applied for haploid breeding, but it also is an ideal in vitro system for investigating cell fate determination and plant embryogenesis. Epigenetic reprogramming is critical for the initiation of microspore embryogenesis, which appears to be similar to somatic embryogenesis. It worth comparing the mechanisms behinds the different roads to embryogenesis, including zygotic embryogenesis, microspore embryogenesis, and somatic embryogenesis, in further studies. In addition, as the single-cell epigenome sequencing technologies develop, profiling DNA modifications and histone modifications during the initial stages of microspore embryogenesis will greatly aid us in understanding the mechanisms behind microspore reprogramming and haploid embryogenesis initiation.

The studies of in vivo haploid embryogenesis in flowering plants greatly promote the use of haploid technology in plant breeding, especially for maize. Based on the current in vivo haploid induction system discussed above, haploid embryogenesis may be induced by uniparental centromere defects, sperm chromosome fragmentation, and the ectopic expression of transcriptional factors related to embryo initiation in egg cells. These genes responsible for haploid induction are closely related to plant reproduction and, in particular, gamete development and fertilization. For example, *MTL*, *POD65*, *DMP,* and *PLD3* are involved male gametophyte development, *ECS* is involved in egg cell development, and *BBM* and *PAR* are involved in embryogenesis initiation. In addition to haploid embryogenesis, sperm-central cell fertilization to form endosperm is necessary for haploid generation. Thus, basic studies related to the molecular and cellular mechanisms for gamete development, fertilization, and embryogenesis initiation will greatly aid us in establishing or optimizing haploid indcution systems.

At present, there are still several questions that remain to be answered about these haploid induction systems. First, a high haploid induction rate is critical for their applications in plant breeding. Presently, in vivo haploid induction systems are typically accompanied by a high frequency of seed abortion. Reducing seed abortion and increasing haploid induction efficiency are key points for the improvement of in vivo haploid induction systems. Second, haploid induction systems have been established for only a few species, primarily for grass and Arabidopsis. Expanding these haploid induction systems to other important economic crops must be investigated with great care. Thus, more attention should be paid to the mechanisms of haploid embryogenesis, which will not only aid us in understanding haploid embryo formation, but it will also help us to optimize these haploid induction systems and expand the applications of haploid technology in crop breeding.

## Figures and Tables

**Table 1:** List of in vivo haploid systems tested in various species.

Gene	Species	Type of Induction System	Reference
*CENH3*	*Arabidopsis thaliana*	*GFP*-*CENH3*; *GFP*-*tailswap*	[53,106]
	*Arabidopsis thaliana*	Point mutation	[39,42,43]
	*Arabidopsis thaliana*	*BnCENH3*; *LoCENH3*; *ZmCENH3*	[41]
	*Arabidopsis thaliana*	CRISPR/cas9 mutant	[39,79]
	*Zea mays*	*GFP*-*tailswap*	[107]
	*Zea mays*	CRISPR/cas9 mutant	[55]
	*Solanum lycopersicum*	Point mutation	[49]
	*Oryza sativa*	Point mutation	[49]
	*Cucumus sativus* L.	Point mutation	[49]
	*Cucumis melo* L.	Point mutation	[49]
*MTL*/*PLA1*/*NLD*	*Zea mays*	CRISPR/cas9 mutant	[58,59,60,79,102]
	*Oryza sativa*	TILLING	[101]
	*Triticum aestivum*	CRISPR/cas9 mutant	[80,103]
	*Setaria italica*	CRISPR/cas9 mutant	[104]
*DMP*	*Zea mays*	CRISPR/cas9 mutant	[65]
	*Arabidopsis thaliana*	CRISPR/cas9 mutant	[105]
	*Brassica napus*	CRISPR/cas9 mutant	[66,67]
	*Brassica oleracea*	CRISPR/cas9 mutant	[68]
	*Nicotiana tabacum*	CRISPR/cas9 mutant	[67,70]
	*Medicago truncatula*	CRISPR/cas9 mutant	[69]
	*Solanum lycopersicum*	CRISPR/cas9 mutant	[71]
	*Solanum tuberosum* L.	CRISPR/cas9 mutant	[72]
*PLD3*	*Zea mays*	CRISPR/cas9 mutant	[73]
*POD65*	*Zea mays*	CRISPR/cas9 mutant	[74]
*ECS1*/*ECS2*	*Arabidopsis thaliana*	T-DNA mutant	[76,77]
*BBM*	*Pennisetu glaucum*	*PsASGR*-*BBML*	[93]
	*Zea mays*	*PsASGR*-*BBML*	[94]
	*Nicotiana tabacum*	*PsASGR*-*BBML*	[97]
	*Oryza sativa*	*PsASGR*-*BBML*	[94]
	*Oryza sativa*	*pDD45*::*SiBBM1-3*	[98]
	*Oryza sativa*	*pDD45*::*OsBBM*	[96]
	*Ceratopteris richardii*	*35S*::*BnBBM*	[95]
	*Arabidopsis thaliana*	*pDD45*::*BnBBM*	[99]
	*Brassica Napus*	*pDD45*::*BnBBM*	[99]
	*Solanum Lycopersicon*	*pDD45*::*BnBBM*	[99]
*PAR*	*Lactuca sativa*	*pEC1*::*ToPAR*	[100]

## Data Availability

Not applicable.
